# Time-Out with Young Children: A Parent-Child Interaction Therapy (PCIT) Practitioner Review

**DOI:** 10.3390/ijerph19010145

**Published:** 2021-12-23

**Authors:** Melanie J. Woodfield, Irene Brodd, Sarah E. Hetrick

**Affiliations:** 1 The Werry Centre, Department of Psychological Medicine, University of Auckland, Auckland 1023, New Zealand; s.hetrick@auckland.ac.nz; 2 Auckland District Health Board, Auckland 1023, New Zealand; 3 Centre for Children, Families and Communities, Department of Psychology, Central Michigan University, Mount Pleasant, MI 48859, USA; IreneBrodd@yahoo.com; 4 Centre for Youth Mental Health, University of Melbourne, Parkville, VIC 3010, Australia

**Keywords:** time-out, attachment, practitioner review, attributions, parent training, behavioral parent training, parent management training, PCIT

## Abstract

Time-out is a component of many evidence-based parent training programmes for the treatment of childhood conduct problems. Existing comprehensive reviews suggest that time-out is both safe and effective when used predictably, infrequently, calmly and as one component of a collection of parenting strategies—i.e., when utilised in the manner advocated by most parent training programmes. However, this research evidence has been largely oriented towards the academic community and is often in conflict with the widespread misinformation about time-out within communities of parents, and within groups of treatment practitioners. This dissonance has the potential to undermine the dissemination and implementation of an effective suite of treatments for common and disabling childhood conditions. The parent-practitioner relationship is integral to the success of Parent-Child Interaction Therapy (PCIT), an evidence-based treatment which involves live coaching of parent(s) with their young child(ren). Yet this relationship, and practitioner perspectives, attitudes and values as they relate to time-out, are often overlooked. This practitioner review explores the dynamics of the parent-practitioner relationship as they apply to the teaching and coaching of time-out to parents. It also acknowledges factors within the clinical setting that impact on time-out’s use, such as the views of administrators and professional colleagues. The paper is oriented toward practitioners of PCIT but is of relevance to all providers of parent training interventions for young children.

## 1. Introduction

Parent training—also known as Behavioural Parent Training or Parent Management Training—is a term used to describe an empirically sound suite of programmes for the treatment of childhood conduct problems and other childhood psychopathology [1]. Internationally, childhood conduct problems represent one of the most common mental disorders diagnosed in children under seven years [2] and if left untreated, may persist into adulthood with widespread social and economic consequences [3]. Parent training has a more extensive evidence base than any other psychosocial treatment for any disorder in the child mental health context [1]. Prominent examples include the Community Parent Education Program (COPE; [4]), Defiant Children [5], Helping the Noncompliant Child [6], The Incredible Years [7], Triple P [8], and Parent-Child Interaction Therapy ([PCIT; [9]). These programmes are drawn from the work of Constance Hanf and Gerald Patterson in the 1960s and involve two phases—initially strengthening the parent-child relationship through child-led play, and later providing parents with support to have developmentally appropriate expectations of children and manage their children’s challenging behaviour safely and effectively [10,11]. Within Parent-Child Interaction Therapy (PCIT), these phases are known as Child Directed Interaction (CDI) and Parent Directed Interaction (PDI), respectively.

Time-out (technically, time-out from positive reinforcement) involves a brief pre-planned withdrawal of parental attention (typically while the parent remains in the room) and restriction of access to desirable items such as toys, in response to a child’s defiance or non-compliance with a parent’s clear and fair instruction. It is incorporated in the second phase of almost all of the prominent, evidence-based parent training interventions. The intention of these programmes is to equip parents with a range of techniques or strategies to respond to children’s non-compliance or defiance in a safe and effective way. Within these parent training interventions, time-out is introduced alongside teaching parents how to give effective, developmentally sensitive commands; how to use planned ignoring in conjunction with praising the ‘positive opposite’ of an undesirable behaviour; using natural or logical consequences, and other developmentally appropriate ways of responding to a child’s non-compliance or defiance [9]. As such, time-out is one component of a collection of behaviour management strategies, which are predicated on initially strengthening and consolidating the parent-child relationship [12].

Of all of the components that make up parent training programmes, time-out is perhaps the technique that is the most well studied [13]. It appears to be particularly important for parent training programmes that are treating emerging and/or established child conduct problems (as opposed to general parenting advice aimed at preventing difficulties from occurring) [14]. Several recent reviews provide a useful and comprehensive overview of the empirical literature on time-out [12,15,16,17], including observation that “there is no empirical evidence for iatrogenic or harmful effects of time-out” [13].

Yet despite this empirical evidence of time-out’s safety when used appropriately, the strategy remains one of the more divisive and technically challenging parenting techniques. In recent years there has been growing public concern around the safety and appropriateness of time-out [12,18], fuelled by articles in popular press publications and online material; these claims have been described as “wild and unsubstantiated, yet highly visible” [19].

### 1.1. Aims and Scope of This Review

The intention of this practitioner review is not to replicate recent reviews of the empirical literature, nor to present a ‘how to’ guide to using time-out. These are available in the aforementioned published reviews and in treatment protocols, respectively. Rather, the intention is to consider the milieu within which time-out is situated in the clinical or treatment setting, and to highlight aspects of the literature that are of relevance to providers—a style of review which has been described as a practitioner review elsewhere e.g., [20]. It is a targeted review of the literature, that is intended to be informative and accessible, rather than an exhaustive synthesis. It is important to acknowledge the limitations of this style of review, which is not systematic and does not critically appraise the quality of the research literature relating to time-out, and therefore should not be considered a definitive summary. Given that several recent high-quality reviews relating to time-out have been conducted, this paper aims to distil and apply these findings to the clinical context, with specific reference to time-out within PCIT. It aims to make the research literature accessible to and engaging for practitioners. It ultimately aims to consider how practitioners might maintain delivery of, and advocacy for this well-studied component of parent training, in the context of public concern.

### 1.2. Structure of the Review

**Figure 1:**
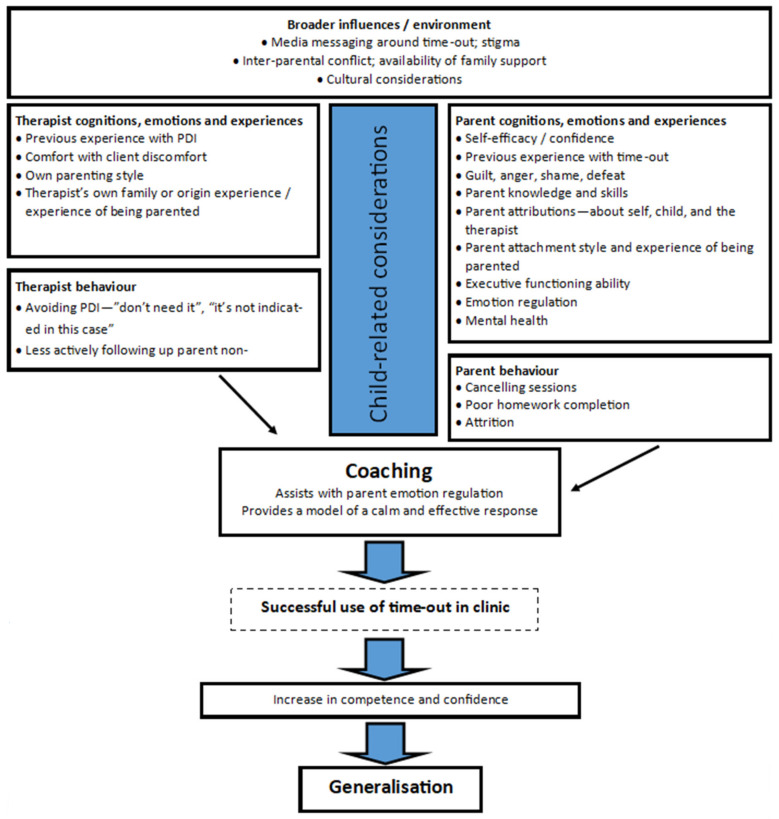
Summary of parent-practitioner related considerations.

Therapists charged with delivery of parent training approaches that include time-out benefit from an awareness of the broader context, including parents’ beliefs and preferences [18]. The review begins with an overview of these wider influences. In clinical settings, time-out is typically introduced, taught, and—within PCIT—coached in the context of a therapeutic relationship between a parent (or caregiver) and practitioner. As such, the review goes on to explore both therapist and parent cognitions, emotions and behaviour, and how these inter-relate. The therapeutic coaching relationship, and the role and influence of factors such as practitioner attitudes toward time-out, transference and counter-transference, and how these elements might influence parents’ ability and willingness to use time-out is also considered. Finally, the review concludes with a series of specific sections relating to (1) Child-related considerations, (2) Addressing specific parent concerns (including the use of ‘time-in’) and (3) Addressing concerns from colleagues or administrators (including a comment on seclusion). The structure of the review is represented in Figure 1. The discussion is oriented around PCIT, though is relevant to providers of other parent training programmes. 

## 2. Broader Influences/Environment

*“[My mother], she’d read a book and she had an idea that… putting Emma on the time-out chair wasn’t a good idea… so there was a real… disorder between the PCIT and at home...” [21].*

Outside of the therapy room, there are a number of influences that may shape the lens through which a parent views time-out. A parent is typically a member of a number of different systems or groups, which may have mixed or disparate views on time-out—for example, extended family, social groups, childcare centres or antenatal groups. Parental stress levels, socioeconomic factors, and the extent of wider family support (including the degree of unity or conflict between parents) are influential on engagement generally [22], and potentially on the acceptability of time-out specifically.

Cultural factors are also very relevant, as these may influence gender roles, parenting styles, and parent engagement in treatment programmes [23]. The research literature relating to the acceptability of time-out to parents (and children) of minority ethnicities is limited [24]. Reviews that have been published have tended to explore the interaction between majority cultural groups and parent training programmes generally [17], or the international transportability of programmes, i.e., whether the programme is still effective when introduced to a different country (e.g., [25]). Relevant to time-out is the extent to which particular cultural beliefs value interdependence, hold that a parent ought to or should assume control of/‘take charge’ of a child’s behaviour, demonstrate affection, and the parent’s level of comfort with limit setting [17].

Often, parent training approaches include techniques that have been developed and normed within an Anglo-American cultural context in the United States [17,24]. This somewhat individualistic (vs. collectivistic) cultural context often values parental control, but also allows for the child to negotiate or reason with their parent, i.e., also values the child’s autonomy and individual freedom [17,24]. If this cultural group is assumed to be the “default”, the advice drawn from parent training programmes may be viewed with distrust by parents from minority cultures, and there may be a dissonance with parent attitudes and beliefs in the diverse real world of service delivery [24]. Future research ought to consider the influence of more precise factors such as families’ acculturation, immigration experiences, and socioeconomic status [24]. Ideally, practitioners ought to facilitate discussion around the family’s religion, family traditions, parents’ own experiences of having been parented [24], and explore how time-out ‘sits with’ the family in relation to their cultural values [17].

Media messaging and public and professional dialogue conspicuously feature two inter-related concerns about time-out, namely, that it (1) causes harm in otherwise healthy children, and that it (2) exacerbates existing difficulties in children who have experienced trauma, despite evidence to the contrary on both counts [12]. Parents are beginning to echo and amplify high-profile media criticisms of time-out, contributing to perception that it is ineffective and harmful [18]. In the clinical context, understanding and addressing parental concerns is essential, as—in terms of parent engagement—the empirical evidence relating to whether time-out causes harm *is perhaps less relevant than a parent’s concern that it might*. Even where parents are weary or ambivalent, fearful of (or angry at) their child, they typically want to do what is best for their child. The therapist-parent relationship is an essential vehicle for validation of parents’ emotion, and an opportunity to provide brief tailored support for the parent as they navigate the often-wide-ranging views and perspectives of the people in their world. 

## 3. Therapist Cognitions, Emotions, Experiences and Behaviour

*“I come from an attachment framework and struggle with some of the aspects of PCIT” (PCIT Practitioner) [26].*

Unless a treatment programme is delivered by way of pre-recorded material (for example, Triple P Online [27]), time-out is typically introduced in the context of a practitioner-parent relationship. A practitioner’s experience with teaching and coaching time-out, the nature of their relationship with the parent and child, and their own level of comfort with client discomfort (in the service of greater goals) may influence their willingness to implement time-out in PCIT. Factors such as the practitioner’s own family of origin experiences (i.e., experience of having been parented) and their own parenting practice (i.e., use of, and attitudes toward time-out with their own children) may also be relevant. Practitioners may underestimate the influence of their own emotional state—perhaps ambivalence or wariness relating to time-out—on their behaviour in session. This is a cognitive bias known as the hot-cold empathy gap that is increasingly considered relevant to the implementation of psychosocial interventions [28]. This dynamic is apparent in another treatment, namely exposure-based tasks within Cognitive Behavioural Therapy for anxiety disorders. Relative to other techniques, exposure tasks tend to be infrequently used by clinicians, with Deacon and Farrell [29] suggesting that this cannot be explained by dissemination difficulties alone. Instead, they propose that “negative beliefs about exposure therapy (e.g., that it is unethical, intolerable and unsafe) impede the utilization of this treatment, even among therapists trained to administer it” [29].

A particular transferential dynamic can evolve in the parent-therapist relationship when a parent raises concerns around time-out. Hawes and Dadds [30] describe this as “the spread of anxiety or pessimism from parent to therapist” (p. 6). Therapists themselves may be somewhat ambivalent about time-out [26], or perhaps anxious about their ability to successfully coach a parent through the early PDI sessions, which can be complex and demanding to facilitate. The therapist may inadvertently conceptualise the parent’s position as resistant, or unconsciously form a rationale for omitting PDI from the PCIT protocol (e.g., that the child’s behaviour has improved substantially in CDI, so PDI is unnecessary), thereby “inadvertently collud[ing] to avoid strategies that require parents to set limits on misbehaviour” [30]. Recognising and becoming aware of these dynamics is an essential step in addressing these common “signs of a struggle for change” [30].

The early attachment experiences of both the therapist and the parent may manifest in the therapeutic relationship during the course of a parent training intervention. Core sensitivities are internal working models, anxieties or considerations that an individual holds in relation to their role in connection with others [31,32]. While these dynamics are primarily conceptual rather than empirical, they can be useful in assisting with understanding a particular pattern of connection between therapist and parent. For example, an *esteem sensitive* parent may strive to demonstrate success or achievement (e.g., with homework completion) and may be very vigilant and sensitive around criticism (e.g., in coaching, which is typically more directive in PDI) [32]. A *separation sensitive* parent may experience limit setting as conflict, which is potentially associated with separation [31]—for this parent, taking charge during the PDI phase of PCIT may be particularly challenging and require additional therapist support. At its best, the parent-practitioner relationship can provide a safe haven and secure base for the parent, and a model of what “bigger, stronger, wiser and kind” looks and feels like to a child [32].

## 4. Parent Cognitions, Emotions, Experiences and Behaviour

*“…while [PDI sessions] were horrible sessions, in many regards, they were the most valuable sessions because it taught me what I could do with him under many situations and recover the situation and not let my child ruin my life. And not let him have… parents that didn’t like him” [21].*

In recent years, and in the context of increased media and public concerns relating to time-out, three studies have investigated parents’ understanding of time-out [33,34,35]. Findings across all three studies demonstrated that parents’ understanding of the purpose and procedure for time-out differed from the empirical literature. The majority of parents perceived time-out as a time for their children to “think” [34] “think about bad behavior” [35], or “think about what they had done” [33]. This is in contrast to the theoretical rationale for time-out, i.e., to remove the child from a reinforcing environment following misbehaviour [12]. Beyond their technical understanding of the time-out process, a parent’s acceptance of a discipline technique such as time-out includes measures of their willingness to use it with their own child, anticipated disruption of implementing the discipline, perceived effectiveness of the technique, and expectation that using the discipline would lead to improvement in their child’s behaviour [36]. Parents who reported using time-out with their 1-to 10-year-old children and rated it as being effective were significantly more likely to report using empirically supported time-out steps [35]. Also, expected relationships emerged between parents’ understanding about time-out, their use of time-out with their own child, and their acceptance of the technique [33]. Parents who endorsed accurate knowledge about time-out rated an evidence-based description of time-out as more acceptable than parents who endorsed less accurate knowledge. In contrast, parents who endorsed more negative attitudes and beliefs about time-out perceived an evidence-based description of time-out as less acceptable than parents who endorsed fewer negative attitudes. Beyond ratings of acceptability, parents’ accurate understanding as well as negative attitudes and beliefs about time-out were significantly associated with their use of empirically supported time-out steps. Parents who agreed with accurate beliefs about the safety and effectiveness of time-out were more likely to report using a greater number of evidence-based time-out steps when using time-out with their children. In contrast, parents who indicated holding more negative attitudes toward time-out were more likely to report using fewer evidence-based time-out steps [33].

This interplay of parents’ experience using time-out with their children, their understanding about time-out, and their perceptions of the effectiveness of time-out are likely all coming into the treatment room when they meet with the therapist. The parent may also be experiencing feelings of inadequacy, overwhelm or anxiety, anger, guilt or shame from a ‘history of 10,000 defeats’ in disciplinary interactions with their child [37].

Time-out is first mentioned early in the course of PCIT in the intake assessment, where therapists are encouraged to “ask specifically” about a parent’s use of timeout [9], p. 11. In our experience, parents often respond with words to the effect of “I’ve tried time-out, but it didn’t work”. A distinction may be drawn between a parent’s *experience* of time-out having been ineffective, and their *perception* that it would be ineffective for their child. Each of these scenarios might require a tailored response from the PCIT therapist, as described below. 

For example, prior to using time-out, a parent might form an *impression or perception* that time-out would be ineffective for their child, perhaps partly as a result of their child-referent attributions or cognitions around the cause of their child’s disruptive behaviour. These causal explanations for a child’s challenging behaviour and cognitions about their parenting role, that parents form implicitly or explicitly may influence how parents engage with parent training and may predict attrition from treatment [38,39,40]. For example, if a parent’s attributions suggest that the cause of the child’s difficulties is internal to the child and stable, this is likely to influence their willingness to consider changing their own behaviour and engaging with a technique such as time-out—stated plainly, there may be an immediate sense of “that won’t work—he’s a bad kid”. The parent may form the impression that time-out is not novel, sophisticated or salient enough to change the behaviour of a child who is perceived to be manipulative, vindictive or deviant. In response, the PCIT therapist might name or describe the apparent dissonance between a parent’s sense of what the child needs and what PCIT is advocating and spend more time explaining the rationale.

As outlined earlier, it is also possible that a parent has *experienced* time-out as ineffective in the past, as it can be difficult to implement correctly [13]. Time-out is not one technique, but a series of steps, that are ideally implemented sequentially and in a pre-determined order (refer to Table 1 for these components and their associated evidence and the Supplementary Material for a case vignette). Omitting or substituting one or more components of the time-out process or applying time-out inconsistently, may inadvertently worsen a child’s disruptive behaviour [41], potentially discouraging a parent from using time-out again, and fostering a perception that it is ineffective.

Often, the parent comes to PCIT having inadvertently established a pattern where aversive discipline interactions with their child are occurring regularly and are rich in content which relates to basic attachment needs in the child [61]. Positive parent-child interactions have become less frequent and typically have become “attachment neutral” [61]. If reward strategies such as star charts or labelled praise are infrequent and ‘neutral’, and discipline interactions are frequent and ‘rich’, it is easy to see why a parent might perceive that time-out is ineffective [61]. Time-out may remain “subtly infused with attachment-rich behaviours (e.g., hostility, rejection, ambivalence) that are highly salient and threatening to the child” [61]. Successful use of time-out depends on the parent shifting the balance, to ensure that positive or neutral time with their child is richer (from an attachment perspective) than disciplinary exchanges. Therapist coaching in PCIT is particularly well placed to ensure this occurs – the coach may encourage the parent to use positive voice tone, physical touch, eye contact, and expressions of enjoyment in both CDI and PDI. 

## 5. Coaching Time-Out

*“[The coaches] talked me through it very calmly, just with their calmness of their voice, sticking to the plan, and I guess as outsiders and probably that division of the glass as the outsiders looking in, they’re not in the heat of the moment, and so they talked me through the heat of the moment… what would have been impossible at home” [21].*

The decision that a parent makes, in the moment, of which strategy to use in response to their child’s challenging behaviour is often instinctive, rather than intellectual. Many parents are able to describe the optimal response to a child’s behaviour hypothetically, however ‘real world’ parenting behaviour involves the interaction of a number of complex processes. These include a parent regulating their arousal levels and emotions and demonstrating inhibition and self-control [38,40]. These processes are particularly relevant to the PDI phase, and specifically to the use of time-out, as disciplinary exchanges can be provocative for parents [62]. PCIT and other programmes that include in vivo coaching of parents with their child offer a distinctive opportunity to rehearse and consolidate alternative responses, alongside the coach who provides social modelling of a calm and effective response [21]. With repetition, a process of ‘overlearning’ occurs, facilitating the easier recall and use of the strategy when required in the real world [63]. As such, coaching is important for parent skill acquisition [64], but it appears to serve a number of additional functions for the parent. For example, recent research with child welfare-involved families suggests that PCIT supports the development of parents’ inhibitory control and emotion regulation abilities, and the softening of negative attributions about their child [40]. A recent paper proposes a model of how this change comes about, with parent coaching as a central mechanism of change, including suggestions that the PCIT coach provides “real time regulatory support” for the parent [65].

All coaching is not created equal, however. Directive techniques (i.e., telling the parent what to do) may inadvertently contribute to parent resistance; alternatively, responsive coaching appears to be particularly useful for parents’ skill acquisition and engagement [66]. Positive, responsive coaching reinforces parents’ use of a particular technique or interaction with their child [66]. Examples of responsive coaching techniques include providing labelled or unlabelled praise for parents’ behaviour or interactions with their child or linking the child’s behaviour to the parents’ use of skills [66]. The PCIT coach can also subtly interrupt a parent’s harsh response, and a potentially coercive parent-child exchange, and support the parent to generate an alternative response in the moment [65]. 

Coaching a parent to use time-out effectively may also assist a parent who has previously felt ineffective or ill-equipped to experience a sense of mastery or competence. An anxious parent has an opportunity to be exposed, with the support of the PCIT coach, to that which they fear, i.e., their child’s defiance or non-compliance. Repeated experiences of successfully managing this process will likely build the parent’s confidence and decrease avoidance of limit-setting.

## 6. Child-Related Considerations

The standard PCIT time-out procedure is indicated for children with a developmental age of approximately 2.5 years and above [9]. For developmental and relational reasons, time-out is not indicated for children younger than two [67] and for practical reasons, other strategies (such as incentive systems or removal of privileges) are typically used with older children [68]. A specific adaptation has been developed for toddlers younger than 2 years old, where the follow-up after a command involves a guided compliance procedure, i.e., the parent gently guides the child in following their instruction; [67]. The adaptation assumes that toddlers have not yet achieved the required language comprehension, ability to sustain attention, and social awareness to comply with parent instructions [67]. Studies have also described adaptations to the PCIT protocol—typically for younger children—that do not involve the use of time-out, including Parent-Child Attunement Therapy [69].

Similarly, an adaptation to the standard procedure has been developed for children on the Autism Spectrum [70]. It includes a time-out readiness phase where there are concerns around the child’s language comprehension, extreme behaviours (e.g., self-injury, extreme aggression), or relating to parent reluctance to use time-out with their child with special needs [70]. The adaptation also includes a physical guidance contingency for rapidly and effectively concluding the time-out process where necessary, which the authors describe as the Big Red Stop Button [70]. These examples of adaptations acknowledge the importance of considering factors such as the child’s age, cognitive abilities, and adaptive skills.

There is little published research on the acceptability of time-out to the child, and the child’s experience of time-out. During a time-out process, children may shout, scream, hit, kick or cry, and it is often assumed that this represents a time of distress for the child—a frequently cited critique of the technique. Another possibility is that—rather than distress—the child is protesting the implementation of new limits and consequences. Learning to stay on a time-out chair as a pre-explained consequence for non-compliance with a calm, fair and reasonable command, represents a series of small and repeated challenges for the child, and this may be conceptualised as an opportunity to develop resilience and self-regulation. Also, experiencing mild or moderate, short-lived anger, frustration or anxiety may be important in helping children develop emotion and behaviour regulation skills [19]. Importantly, the child learns

*“no matter how upset I am, no matter how much I cry, scream, kick, or shout curse words, I will be safe. No one will yell at me or hit me. My parents will remain regulated” [19].*

For the child, time-out may represent a safer alternative than physical discipline, as a disciplinary exchange can represent a period of higher risk of physical harm for the child [40]. Unlike spanking, brief time-outs can be used several times per day initially (their required frequency would be expected to decrease rapidly if implemented correctly), which allows the parent to be more consistent in their response [68].

## 7. Addressing Specific Parent Concerns

If a parent raises doubts, concerns or questions about time-out, it can be useful to initially thank the parent for doing so [30]. It is possible that the parent is posing a question that can be addressed by providing specific information or recommending a resource—indeed, one of the functions of parent training programmes is to support parents to discern which skill, strategy or response is indicated in a particular scenario. Niec [19] provides a compendium of possible therapist responses to specific parent concerns.

However, oftentimes there is a deeper concern or process that might be occurring for a parent. For example, a parent asking, “What’s the evidence for time-out, anyway?” is unlikely to be requesting a precis of the latest meta-analysis but may in fact be wondering “Am I doing the right thing for my child?”. Similarly, a parent who appears resistant (perhaps by persistently replying “yes, but…”) may have a deeper need that relates more to the *process* of therapy, rather than the *content* under discussion. Naming or reframing this can be useful, as “resistance that is implicit and unspoken is at particular risk of continuing unchecked. Naming it allows the therapeutic team to examine it openly together” [30]. An example of naming this process might include “I can provide you with evidence if that would be helpful, though I wonder if perhaps you have deeper concerns about this stage of the programme”. A reflective statement, followed by a pause or silence from the therapist, can result in a parent sharing fears or concerns that can then be more usefully addressed.

Another possibility is to acknowledge the parent’s ambivalence around time-out, followed by the conjunction “and” (cf. “but”)—for example: “I’m aware that you’ve used time-out in the past and have had limited success, and I have a specific way of doing time-out to share with you that I’m confident will be effective”. This conjunction substitution is utilised in Dialectical Behaviour Therapy (DBT; [71]) and can signal that both perspectives have validity. In this case, it serves the purpose of validating the parent’s experience, while also indicating that a different experience is possible with time-out. There is growing interest in improving parent emotion regulation through integration of DBT principles within parent training [72]—as outlined earlier, this may be achieved formally, or through the naturalistic opportunities afforded by the coaching relationship between parent and therapist in PCIT [65].

There are certainly situations where time-out is not indicated and may indeed be ineffective or contraindicated. The PCIT protocol recommends time-out as a response to a child’s non-compliance with an effective command (i.e., a direct, necessary, developmentally appropriate parental instruction), and suggests that commands are to be used sparingly [9]. When a child is distressed or overwhelmed (perhaps due to an injury, tiredness or hunger), choosing not to give a command, but rather attending to the primary need, is a more suitable response than time-out. Likewise, behaviour such as whining or complaining ought to ideally be responded to with brief planned ignoring, rather than time-out [9]. Time-out is not indicated as a response to a child having a tantrum. Where a young child is struggling to regulate their emotions outside of the parent giving directions, or the child is tired or hungry, a parent providing a “time-in” (as described below) is likely to be the most appropriate response. Table 2 provides examples of how these scenarios may be differentiated. Lieneman and McNeil [17] also present a useful table to assist parents to determine whether it is an appropriate time to use a command (which may go on to require time-out if the child is non-compliant), along with a summary of alternatives to commands. They suggest that in order for a direct command to be used, a child must be well-rested, not be too hungry or thirsty, be alert, have recently used the toilet, and be ready to learn [17].

### Time-In

Time-in is inconsistently defined in the academic literature and popular press. One understanding of time-in is aligned with a parent providing the PRIDE skills (Praise, Reflection, Imitation, behavioural Description, and Enjoyment) in the Child Directed Interaction phase of PCIT. Use of these skills is recommended any time the child is not in time-out. When a child experiences intense disappointment, anger or frustration that is not necessarily associated with aggression or destructive behaviour, sitting alongside the child and describing their experience can aid the development of their emotion regulation abilities [73]. Paired with differential attention, the parent models calmness through their tone of voice and attends to their child’s appropriate behaviour. Recognising a child’s emotion, labelling this, and validating their experience (not necessarily their actions) can enhance children’s social and emotional functioning [74].

## 8. Addressing Concerns from Colleagues or Administrators

*“I have become uncomfortable about the use of time-out by PCIT. For the children I see with trauma histories—this is entirely inappropriate” (PCIT Practitioner) [26].*

### 8.1. Attachment-Based Interventions

The popular perception of a dissonance between behaviourally based and attachment-based paradigms is unhelpful and underpins much of the contradictory material available to parents. In fact, the parent-child attachment relationship is the foundation upon which PCIT stands, as evidenced by CDI being central to the treatment and delivered first. In recent years, attempts have been made to bridge this divide by observing the overlap or commonalities across the two models – for example Troutman’s [75] book “Integrating Behaviorism and Attachment Theory in Parent Coaching”. In this book it is proposed that the ideal parent-child attachment relationship ought to be hierarchical and that a parent being in charge and setting limits is not mutually exclusive with an attachment-oriented approach. Troutman [75] observed that attachment theorists such as Mary Ainsworth—contrary to popular belief – have suggested that a child benefits from “learning about the limits of their power and not being able to control their parents” [75]. Although Ainsworth did observe that the child ought to first control his or her own world through parents responding to their requests [75], as is reflected in the CDI phase of PCIT. Similarly, prominent attachment author Dr Dan Siegel has actually suggested that the use of time-out is reasonable in his statement that “the “appropriate” use of time-outs calls for brief, infrequent, previously explained breaks from an interaction used as part of a thought-out parenting strategy that is followed by positive feedback and connection with a parent. This seems not only reasonable, but it is an overall approach supported by the research as helpful for many children” [76]. Unfortunately, this middle ground, which is likely to represent a position with which many experts agree, has not received the same degree of media attention as the more polarised views of time-out.

Also, perhaps rather than an attachment-focussed intervention being considered superior or inferior to a parent training programme, it may be more useful to consider timing and context. Often parents of children with conduct problems present to services for treatment when they are in crisis. At that time, they are often seeking (and treatment planning typically indicates) a series of evidence-based strategies to make daily life more manageable. Once the crisis has resolved somewhat, the parent is likely to be better able to make use of an attachment-focussed intervention that may involve enhancing reflective functioning and mentalising ability but doesn’t necessarily provide specific strategies. An example might include the Circle of Security Intervention [32], which—in keeping with the middle ground outlined above, advocates for parents to be ‘bigger, stronger, wiser and kind’.

### 8.2. Seclusion

Efforts are underway internationally to reduce agencies’ use of seclusion (i.e., placing and detaining an individual in a room) and restraint (i.e., confining an individual’s bodily movements) as it has been suggested that they can result in significant harm—both physical and psychological [77]. In contrast to provision of other parent training programmes where parents might be advised to rehearse using time-out in their home, PCIT presents a somewhat unique challenge to agencies in that parents are supported to place their child on a time-out chair, and—if necessary—in a time-out room for a one-minute period, on agency premises. However, several elements differentiate the use of time-out from seclusion: (1) rather than a service provider, it is the child’s parent who initiates and carries out the process, and the clinician does not implement time-out with the child; (2) the parent elects to undertake the process and may choose to end the process at any time; and (3) PCIT (and indeed other parent training programmes) include information and support for parents to discern when to use time-out, and when alternative strategies would be indicated.

The issue of agencies seeking to reduce seclusion has been identified as a barrier to implementation at a policy level in large-scale PCIT initiatives in the USA, though examples are available of contexts where difficulties were resolved by way of creating a policy clarification, providing additional education or adapting implementation [78]. In New Zealand, the Ministry of Health issued a position statement in 2019, suggesting “a clear distinction between a clinician coaching a parent to use time-out in a relationally based paradigm and a mental health service using seclusion for safe containment” [79].

## 9. Conclusions

Time-out is not intended to be used as a stand-alone technique in the management of children’s challenging behaviour, but rather as one component of a multi-faceted approach which includes parent-child relationship enhancement as its foundation [17,19,75]. The behaviour management phase of parent training interventions such as PCIT typically includes a variety of components, of which the correct and appropriate use of time-out is but one [17]. Parents are supported to give effective instructions, which are developmentally appropriate, calmly stated, clear, and given one at a time [9]. Importantly, parents are encouraged to use such direct commands sparingly, and to be consistent and fair both in their expectations of their child, and in their use of consequences [9].

This practitioner review, while not an exhaustive or systematic summary of the literature, has identified areas that warrant future research attention. These include a better understanding of professionals’ knowledge of, and attitudes toward time-out, and how this influences the implementation of PCIT in clinical settings. There is also a need for a careful examination of the practitioner-related factors (e.g., education, training, experience, or context) that are associated with effective and sustained implementation of parent training approaches that include time-out. And, importantly, more research into a child’s experience of time-out is also necessary.

In summary, the parent-practitioner relationship is integral to the success of Parent-Child Interaction Therapy (PCIT) yet this relationship, and practitioner perspectives, attitudes and values as they relate to time-out, are often overlooked. Delivering parent training interventions that include time-out can be challenging for practitioners. Misinformation abounds, the technique involves a number of steps, and coaching time-out processes in the clinic can be challenging for practitioners—both practically, and emotionally. Yet, given the effectiveness and established safety of time-out, and the potential harm associated with untreated or ineffectively treated childhood conduct problems, persisting with the delivery of evidence-based parent training programmes which include time-out is likely to result in parents being better equipped to respond to their child’s challenging behaviour effectively, sensitively and safely.


**Key Practitioner Messages**

–The parent-practitioner relationship is central to the success of Parent-Child Interaction Therapy (PCIT) yet this relationship, and practitioner perspectives, attitudes and values as they relate to time-out, are often overlooked.–The intention of parent training programmes is not to advocate for or mandate the use of time-out. Rather, the intention is to enhance a parent’s ability to give fair, reasonable, effective commands when required, and to discern which technique or strategy is indicated as a developmentally ap-propriate response to a child’s non-compliance or defiance.–One discipline technique is not necessarily better than another. Having an understanding of, and competence in using, a range of safe and effective techniques is important for parents of children with conduct problems.–Different children need different responses. Factors such as a child’s age and temperament, severity of the child’s conduct problems, and a parent’s capacity to utilise the technique calmly, consistently and infrequently, are all relevant considerations. Often, children’s behaviour can be effectively managed with other strategies, and time-out is not necessary–Time-out is not a one-size-fits-all technique, and clinical assessment and formulation remain im-portant.


## Figures and Tables

**Table 1:** Time-out components and their associated rationale.

	Description/Definition	Rationale/Research Evidence
One time-out warning	One, brief explanation that time-out will occur if misbehaviour persists.	Children complied with directions when they were warned once that time-out would occur if misbehaviour continued [42]. Children received significantly fewer time-outs when a warning was given than when no warning was given (M = 2 vs. 7 time-outs) [43].
Reason for time-out	Parents identify the misbehaviour that led to time-out.	Children were significantly less disruptive during time-out when parents stated the reason before starting time-out [44].
Time-out location	Select the least restrictive time-out location which minimizes rewarding activities.	Time-out from reinforcement removes a child for a brief period of time from enjoyable activities (e.g., playing with toys, screen devices, talking to others) [35,45].
Initiate time-out	Parents (a) get their children to time-out and (b) set the expectation for children for time-out.	To reduce the amount of attention their children get after misbehaviour, parents should use the least restrictive verbal and/or physical approach necessary to get their children to time-out [41,46,47]. Parents should set an expectation for time-out that is consistent with the purpose of time-out (e.g., “stay in time-out” until the parents give permission for the child to leave) rather than commonly mis-used expectations for the child to calm down or think about what they did [34,35].
Removal from reinforcement	Remove the child from activities such as playing with toys, screen devices (e.g., tablet, TV, phone) and/or receiving social attention.	Core to time-out is the removal of children from reinforcing or rewarding activities (e.g., play, social interactions) for a short period of time after misbehaviour [48]. Parents should ignore anything their children do (e.g., whine, sass, yell, apologize, negotiate about time-out, etc.), and avoid telling children to be quiet as long as they stay in time-out, because responding would reinforce attention-seeking behaviours, making them more likely to occur in the future [35].
Time-out duration	The minimum amount of time that children must remain in time-out.	A brief, 2- to 3-minute consistent time-out duration should be used for children 2-to-8-years old. Shorter time-out durations are equally or more effective than longer durations [48,49,50,51,52]. The less time children spend in time-out, the more time they spend in rewarding environments where they can learn and be reinforced for desired behaviours [51].
Parental release from time-out	Parents, not children, determine when time-out ends.	When parents, versus the children, determined the end of time-out, preschoolers with disruptive behaviours complied with significantly more of their parents’ directions (78% vs. 44%) and they required significantly fewer time-outs (M = 6.5 vs. M = 16) [53,54].
Contingent release from time-out	Time-out ends after children have stayed in time-out for a minimum length of time and were quiet at the end of time-out.	In comparison to time-based release (i.e., time-out ends after a set amount of time regardless of children’s behaviour), when parents used contingent release, their children were less disruptive during later time-outs [49]. If children are engaging in disruptive behaviours (e.g., yelling, screaming) at the end of the set time-out duration, parents should wait until their children are quiet for a brief time (e.g., 5 seconds), so that they reinforce appropriate behaviour by ending time-out [49,53,55].
Time-out escape contingencies	Parents respond if their children leave time-out before parents give permission	Parents should use the least restrictive escape contingency, e.g., repeated return to chair; [56] before using more restrictive methods (discussed below) to teach children that leaving time-out without permission does not end time-out. Using a time-out room (i.e., a safe, small, well-lit room which the child cannot leave until their parent permits) as an escape contingency led to fewer escape efforts so that only the time-out chair was needed for future time-outs [54,57]. If children’s misbehaviours are severe enough to necessitate the use of a time-out room, parents are encouraged to seek professional support. Previously recommended responses to time-out escape, such as spanking or physically restraining a child to remain in time-out are not recommended [58,59].
Compliance with original directions	If children go to time-out for not following directions, after time-out, children must obey the original directions, or go back to time-out.	If children can go to time-out to avoid doing undesirable tasks (e.g., putting away toys), time-out can reinforce non-compliance [60]. When children were required to comply with their parents’ original directions after time-out, their compliance increased from obeying 60% or fewer parental directions to obeying 70 to 90% of commands [60].

**Table 2:** Examples of scenarios to demonstrate the use of “time in” and “time-out” across a spectrum of behaviour.

Scenario	Indicated Parent Response(s)
Child is emotionally dysregulated in the absence of a direct command—perhaps in the context of limit-setting (e.g., being told “no”) and/or feeling disappointed or frustrated (e.g., another child is using a toy they desire).	“Time-in”—parent moving close to the child and providing PRIDE skills. Parent recognising the child’s emotion, labelling this, and validating their experience (e.g., “I understand you’re disappointed—it’s hard to wait”).
Parent is seeking alternatives to giving directions which could require a time-out.	“When… then…” statements (“when you finish your peas, then you can have yoghurt”) ‘Broken record’ technique of repeatedly stating a brief summary (“screen time has finished for today”). Timed challenges / races to complete the task. Providing a choice where possible (e.g., “do you want a big spoonful of peas on your plate, or a little spoonful of peas?”)
Child is rarely displaying a behaviour that the parent would like to occur more frequently (e.g., being gentle with other people, animals or toys; sharing; completing a specific chore).	Intensive labelled praise of the specific behaviour. Star chart / reward chart to promote a period of intensive reinforcement of a specific behaviour.
Child is irritable or oppositional in the context of being tired, overwhelmed, hungry or in pain.	First, address the primary need where possible. Brief planned ignoring, coupled with immediate praise for the ‘positive opposite’ behaviour. Deliberate decision not to give a command (which could result in needing to use time-out if the child does not comply). Distraction.
Child is non-compliant with a direct, effectively stated, reasonable command or instruction (e.g., “We’re going to Grandma’s house. Please bring me your shoes”).	Time-out (as described in Table 1 above).

## Data Availability

Not applicable.

## Supplementary Materials

The following are available online https://www.mdpi.com/article/10.3390/ijerph19010145/s1, Box 1: Vignette illustrating an evidence-based time-out process for non-compliance with a parent’s instruction.
