# Supplementary material for: Time-Out with Young Children: A Parent-Child Interaction Therapy (PCIT) Practitioner Review
Source: Int J Environ Res Public Health. 2021 Dec 23;19(1):145. doi: 10.3390/ijerph19010145 (PMC8750921; doi:10.3390/ijerph19010145)
Supplement: Supplementary file 1 [file ijerph-19-00145-s001.zip › ijerph-1495114-supplementary.pdf]

In the evening, 5-year-old Taylor is in the living room playing with Lego while her parents, Mr. Johnson and Ms. Johnson, are preparing dinner. Mr. Johnson approaches Taylor and says "it's almost time to eat. Please put your Lego in the box." Taylor whines that she is not hungry and wants to keep playing. Mr. Johnson remains quiet for a brief period (i.e., 5 seconds) to allow Taylor time to start following his directions despite her protests. When she continues to build, Mr. Johnson gives a **time-out warning**: "*Taylor, if you don't put the Legos in the box, you will have to sit on the time-out chair.*" Mr. Johnson points to the chair that is in the corner of the living room. He again waits briefly (i.e., 5 seconds) to allow Taylor to start obeying. She continues to whine. Mr. Johnson calmly places his hand around Taylor's shoulder while saying the **reason for time-out**: "*because you didn't do what I told you to do, you have to go to the time-out chair*." Taylor huffs and stands up.

Mr. Johnson walks beside her to the **time-out location**. The time-out chair is a sturdy, adult-sized chair in the corner of the living room, away from toys, books, or screen entertainment (e.g., TV, tablets). Mr. and Ms. Johnson are able to supervise Taylor from the kitchen while they continue to prepare dinner. Mr. Johnson **initiates time-out** by telling Taylor, "*stay in time-out until I say you can get off*." Taylor huffs and protests that she does not want to be in time-out, but remains seated in the time-out chair. By ignoring her whining and avoiding further explanations about why she is in time-out, Mr. Johnson maintains the safe but boring time-out environment by **removing reinforcement**. Mr. Johnson looks at the wall clock and notes when the minimum **time-out duration** (i.e., 3 minutes) will end. Taylor continues to whine that she is not hungry and doesn't want to eat. Since she does not receive attention for this, after several minutes she rests her chin in her hands and quietly watches her parents prepare dinner. [If Taylor got off the time-out chair before Mr. Johnson permitted, he would respond with a **time-out escape contingency**. He would guide her back to time-out, say "*stay in the time-out chair until I say you can get off,*" and restart timing 3 minutes.] Since Taylor is quiet, when 3 minutes elapses, Mr. Johnson approaches to **release her from time-out.** To reinforce her appropriate (e.g., quiet, calm) behavior, Mr. Johnson says, "*you are sitting quietly in the chair"* (**contingent release from time-out**). He gives Taylor another opportunity to **comply with his original directions** saying, *"are you ready to come back and put the Lego in the box*?" Taylor quietly walks over to the Legos and starts putting them away. Mr. Johnson smiles and her and says, "thank you for doing what I said."

Supplementary Box S1: Vignette illustrating an evidence-based time-out process for non-compliance with a parent's instruction. The 10 step-by-step time-out components are marked in ***bold*** *throughout the text. Examples of wording parents may say for each of the verbalized components are in italics.*
